# *Homo faber* Revisited: Postphenomenology and Material Engagement Theory

**DOI:** 10.1007/s13347-018-0321-7

**Published:** 2018-07-30

**Authors:** Don Ihde, Lambros Malafouris

**Affiliations:** 1 Stony Brook University, Stony Brook, USA; 2 Keble College & Institute of Archaeology, University of Oxford, Oxford, UK

**Keywords:** Making, Materiality, Tools, Embodiment, Evolution, Human becoming, Praxis, Anthropology, Technical mediation, Embodied cognition

## Abstract

Humans, more than any other species, have been altering their paths of development by creating new material forms and by opening up to new possibilities of material engagement. That is, we become constituted through making and using technologies that shape our minds and extend our bodies. We make things which in turn make us. This ongoing dialectic has long been recognised from a deep-time perspective. It also seems natural in the present in view of the ways new materialities and digital ecologies increasingly envelop our everyday life and thinking. Still the basic idea that humans and things are co-constituted continues to challenge us, raising important questions about the place and meaning of materiality and technical change in human life and evolution. This paper bridging perspectives from postphenomenology and Material Engagement Theory (MET) is trying to attain better understanding about these matters. Our emphasis falls specifically on the human predisposition for technological embodiment and creativity. We re-approach the notion Homo faber in a way that, on the one hand, retains the power and value of this notion to signify the primacy of making or creative material engagement in human life and evolution and, on the other hand, reclaims the notion from any misleading connotations of human exceptionalism (other animals make and use tools). In particular, our use of the term *Homo faber *refers to the special place that this ability has in the evolution and development of our species. The difference that makes the difference is not just the fact that we make things. The difference that makes the difference is the recursive effect that the things that we make and our skills of making seem to have on human becoming. We argue that we are *Homo faber *not just because we make things but also because we are made by them.

Humans are no mere creatures of ‘nature’ or ‘biology’. They are not solely the products of ‘culture’ either. Rather, the human mode of being can be better described as ‘a continuum of human-prostheses inter-relations’ (Ihde 2012, 374). That is a mode of being in between the imposed notional frontiers of ‘nature’ and ‘culture’ or ‘mind’ and ‘matter’. Humans achieve this relational ontological standing primarily by means of *creative material engagement* which can be defined as ‘*a long-term commitment to the discovery of new varieties of material forms, so far as it is possible in a given historical situation, through a saturated, situated engagement of thinking and feeling with things and form-generating materials*’ (Malafouris 2014, 144 italics in the original). Such a relational view brings with it a new vision of human becoming (evolutionary and developmental). At the heart of this new vision lies a basic premise: humans more than just adapting to their environments are also actively changing them (for better or worse), initiating new complex co-evolutionary paths and biosocial synergies (Laland et al. 2000; Laland et al. 2014; Laland 2017; Renfrew et al. 2008; Malafouris 2013, 2015, 2016a,b; Ingold 2004; Ingold and Pálsson 2013; Fuentes 2016). Put it more simply: we make things which in turn make us. We use the term ‘things’ in the broad sense of material forms and techniques—it refers to the materiality of mundane objects, tools and artefacts as much as it refers to modern technologies and new forms of digital culture.

This ongoing dialectic between people and things is not something new. It has long been recognised and variously interpreted from different disciplines in the humanities as well as in the cognitive and social sciences. Still the basic and perhaps more radical idea that humans and things are co-constituted or entangled continues to challenge us, raising important questions about the place and meaning of materiality and technical change in human life and evolution that are not yet fully resolved: What exactly do we mean when we say that technologies (old and new) make us just as much as we make technologies? Exactly how are techniques and technological transformations impacting human life and thinking? What is the best approach to the study of this relationship of co-constitution between people and things? To answer those questions, we need a better understanding of technical mediation, which in turn demands better descriptions and cross-disciplinary accounts that would do justice to the complexity of this multifaceted phenomenon of material engagement.

This paper and the special issue of *Philosophy and Technology* that it introduces combine perspectives from the archaeological/anthropological framework of Material Engagement Theory (MET) (Malafouris 2004, 2013; Malafouris and Renfrew 2008, 2010; Renfrew 2004; Gosden and Malafouris 2015), and the philosophical notion of postphenomenology (PP) (Ihde 1979, 1990, 2009; Ihde et al. 2015; Verbeek 2005, 2011; Rosenberger and Verbeek, 2015; Friis and Crease, 2016) trying to attain better understanding about these matters. We believe that Material Engagement Theory and postphenomenology complement each other’s formational and situational interests. The former, rooted in archaeology and anthropology, takes a long-term and comparative view. On the other hand, postphenomenology first took shape in an STS (science and technology studies) context and thus tended to do its case studies on contemporary, often digital and electronic technologies (Verbeek 2008a; Ihde 2009; Rosenberger 2011, 2013). The long view of Material Engagement and the contemporary view of postphenomenology thus complement each other. Each examines the interactivity of different technologies on human developmental experience. Putting aside the disciplinary differences that separate the two approaches (in terms of their respective intellectual traditions, analytical units and temporal scales), in this paper, we would like to highlight what those frameworks share in common and how they can help and complement each other.

In this connection, two major epistemic features that unite postphenomenology and Material Engagement Theory should be stated at the outset:

1. Postphenomenology and Material Engagement Theory share a fundamental commitment to the *relational ontology* in which people and things are inseparably linked (Ihde 1979, 2009; Malafouris 2004, 2013). We change the world and make things that transform the way we experience and make sense of it. We in turn change during this process (Ihde 2009, p. 44). There have been different formulations of this idea in different disciplines, from Marshall McLuhan’s descriptions of media as the extensions of our senses (1994 [1964]) to Bruno Latour’s hybrid networks of ‘actants’ (1993; 1999), to Haraway’s ‘cyborgs’ (1991), to Bernard Stiegler’s ‘originary technicity’ of human existence (Stiegler 1998), to Daniel Miller’s assertions about how things ‘matter’ by means of ‘objectification’ (Miller 1998), to Tim Ingold’s views on material ecologies (2012) and ‘correspondance’ (2013). With postphenomenology and Material Engagement Theory, the emphasis falls specifically on the human predisposition for ‘technological embodiment’ (Ihde 1990) and ‘creative material engagement’ (Malafouris 2014, 2015) as well as on the varieties of skill, praxis and of self-consciousness (Ihde 2009; Malafouris 2008b, 2014, 2015) that come with it. This is unlike anything we see in other animals. Of course, to various degrees, all animals are niche constructors and some of them are prolific users of tools. But the fact remains that no other lifeworld, besides the one we call human, can be argued to be constituted, and thus defined on the basis of its changing relationship with the variety of material objects and technologies that it makes and uses. This statement does not mean to imply discontinuity with other animals (we are all part of the same evolutionary process); it means simply to highlight the special place that fabrication and material culture has in human life and evolution. Wherever technics originated, it is clear that humans expanded and multiplied the interaction—the extravagance of a Tesla Roadster now on a trajectory past Mars illustrates this magnification. The difference we are interested to point out is not one of cognitive ability, brain size or genetic substratum, but refers to the way human self-consciousness is technically and intersubjectively mediated. More than any other animal, humans evolve by creating new materials (from wood, stone and ceramic, through to metals, alloys, glass, paper, concrete, plastics and silicon) and material forms (surfaces, boundaries, lines, containers, houses, wheels, signs, maps, images, letters, documents, machines etc.), and by developing skilled practices opening up to new socio-technical possibilities (sometimes enabling and sometimes disabling).^1^
  Bridging perspectives from postphenomenology and Material Engagement Theory will allow us to revisit and to redefine the term *Homo faber* in a way that can be used to articulate this distinctively creative mode of human becoming. As we discuss in the following section, we adopt the term *Homo faber* because of the emphasis it places on fabrication or making. This emphasis takes us beyond the usual narrow anthropological concern with human or animal capabilities for tool making and tool using. We are using the term *Homo faber* to articulate human-the-maker and not specifically human-the-tool-maker. Although most things can be turned into tools, many things people make are not tools, or in any case they are not used or perceived as such. Think for instance of a dance, of a loaf of bread, or of a line drawn on a piece of paper. Revisiting the notion of *Homo faber* in this paper, our aim is not to argue for the uniqueness of ‘man the tool maker’ but to signify the primacy of making or *creative material engagement* in human life and evolution (Malafouris 2010b, 2012a, 2014; Malafouris 2016a). In particular, our use of the term *Homo faber does not refer to a special ability that only humans have,* rather it refers to *the special place that this ability has in the evolution and development of our species*. The difference that makes the difference is not just the fact that we make things. The difference that makes the difference is the recursive effect that the things that we make and our skills of making seem to have on human becoming (see also Ingold 2013). This view of human becoming is also very different from that which sees the material world as a stock of passive external resources to be exploited. Our vision of *Homo faber* presupposes and actively promotes a parallel vision about the material world as something alive and vibrant (cf. Bennett 2010). That is, we are thinking about technics as an ecology that is inseparably material (Ingold 2012; Knappett and Malafouris 2008a, b), cognitive (Hutchins 2008, 2010, 2011) and embodied (Ihde 2009). The latter point brings us to the second major point of convergence between postphenomenology and Material Engagement Theory.
2. Both theoretical frameworks place the emphasis of their analysis on issues of practice and experience, not on representations. This helps us to collapse the unhelpful opposition between knowing and making or else between cognition and material engagement. Our aim in the following is to challenge this outdated form of thinking about the mind by arguing that such a view which disregards the fundamental structures and features of our engagement with the material world (in the general sense of technics) is unable to account for its operation and evolution. Our understanding of human evolution can never be complete without taking into consideration this process where people and things are inseparably intertwined and co-constituted (Malafouris 2013; Malafouris 2016a,b; Renfrew et al. 2008; Gosden and Malafouris 2015; Knappett 2005; Iliopoulos and Garofoli 2016; Verbeek 2011). This observation follows not just from a long-term archaeological perspective but also in view of the ways new materialities (e.g. digital) increasingly envelop our everyday life and thinking (see Clowes 2015; Clowes 2018; Poulsgaard and Malafouris 2017; Poulsgaard 2017; Ihde 2015). Indeed, the mentioned constitutive intertwining of people and things, of cognition and material culture, was a key dimension in the prehistory of our species, as much as it is active and ongoing in the present. That also implies that there is no ‘core’ or ‘essential’ humanity (biological or other) that pre-exists and which could subsequently be enhanced, extended, disciplined or threatened by technological interventions. Postphenomenology explicitly takes an anti-essentialist, neo-pragmatist shift (Ihde 2016). Technology is at the heart of human becoming but it does not provide or in any sense predetermine a specific direction of change (progressive or other). Humanity has always been inseparable from technical mediation and material engagement. Indeed, a major question that unites postphenomenology and Material Engagement Theory concerns the nature and meaning of ‘technical mediation’ (Latour 1992; Verbeek; 2011) and the primacy of material engagement (Malafouris 2015, 2016a,b, 2018; Ihde 1979). The proposed blending of the postphenomenological perspective on technical mediation with the insights of Material Engagement Theory on the co-evolution of people and things makes it possible to see how humans and things exist in mutual interdependency, beyond the nature and culture distinction.

Based on this shared theoretical foundation we have just described, in what follows, we shall be trying to bring postphenomenology and Material Engagement Theory in closer dialogue with one another hoping to clarify some further issues about the continuously evolving relationship of people with things. We will start with some clarifications of our use and meaning of the term *Homo faber*. Specifically, we want to *rethink* the notion by *unthinking* some persisting assumptions about the nature of continuities and discontinuities between humans and their evolutionary antecedents that seem to misguide our attempt to understand the significance of *fabrication* in human becoming. Then, we extend our discussion in the context of current neo-Darwinian thinking proposing a ‘transactional’ rather than mere ‘interactional’ approach to the meaning of technical mediation. Last, in the final section, we use the example of stone tool making to explore a different set of continuities and discontinuities: those between cognition and material culture or between people and things. We argue against the separatist cognitivist vision of interiorities vs exteriorities and for the primacy of material engagement.

## What Is the Meaning of *Homo faber*?

If we could rid ourselves of all pride, if, to define our species, we kept strictly to what the historic and the prehistoric periods show us to be the constant characteristic of man and of intelligence, we should say not *Homo sapiens*, but *Homo faber*. In short, *intelligence, considered in what seems to be its original feature, is the faculty of manufacturing artificial objects, especially tools to make tools, and of indefinitely varying the manufacture.* (Bergson 1998 [1911], 139)

Bergson was right. Notwithstanding past abuses and oversimplifications in the way the term has been employed, *Homo faber* is a better description of our species than *Homo sapiens*. At first glance, this claim may seem problematic, given all we have learned in recent decades about the tool-using abilities of non-human animals (for good review, see Haslam et al. 2009; Bentley-Condit and Smith 2010; Shew 2017; Shumaker et al. 2011). Indeed, the wrong way to think about the notion of *Homo faber* is to consider tool use and tool making to be an ability limited to the genus *Homo* (see de la Torre 2011). Accumulating comparative evidence and field observations at the interface of primatology and archaeology (Haslam et al. 2009) demonstrates a variety of tool-related behaviours (e.g. stone and plant-material selection, processing and accumulation) in hominins, non-hominin primates and other animals, both in the wild and in captivity. Very recent finds now date tool kits 3.3 Mya, much older than Oldowan tools, pushing tool use to the time of ‘Lucy’ an *Australopithecine*, not previously thought a possible tool user (Harmand et al. 2015). Admittedly, we may be at the dawn of a new narrative where *Homo* is in fact a late-comer to tool use from millennia of such use by pre-*Homo* species.

This recognition however, namely that other animals also habitually accumulate or modify durable materials, well supported by evidence for advanced manual manipulative abilities in pre-*Homo* hominins (Kivell 2015) and by the famous feats of termite-fishing chimpanzees and hook-crafting crows, by no means implies that the notion *Homo faber* lost its power or that this notion should be abandoned. As mentioned, many species make use of tools but no other animal presents anything resembling the immerse variety and complexity of the technical relationships we see in the case of humans. No doubt there is continuity in the way animals build their nests and humans build their houses, or in the way primates select, modify and use a wide variety of plant materials (leaf, wood, twig, grass) for extractive foraging, social interaction and self-maintenance (Haslam et al. 2009). However, there are also important differences that must be given serious consideration before any meaningful comparisons can be made. Though it is both tempting and productive to draw comparisons with the different ways primates and other animals use and engage with their material environments (e.g. using percussive or probe tools to process hidden foods), we must also distinguish ourselves, since we are so different too (Roux and Brill 2005; Tallis 2011; Malafouris 2010c, 2012a).

The claim we wish to make in this paper, captured also in the quote by Bergson, is not a separatist one that views humans as capable of doing something that other animals cannot, namely, make and use tools. Rather, it should be understood as a statement about *how humans become*. Humans are self-conscious fabricators that become (ontogenetically and phylogenetically) through their creative engagement with the material world. Other tool-using animals are not self-conscious fabricators and they do not become by making; they merely manipulate material objects in an extractive foraging context and in predominantly utilitarian fashion. So, we say Bergson was right because he recognised this process of ‘creative evolution’ at the heart of human becoming. That is, he recognised the unique ability of our species to re-create, change and adapt the conditions of our own existence and evolution by means of material engagement. John Dewey, too, interpreted humans as creative problem solvers using an organism-environment model of relationality (Ihde 2016). Dewey’s notion of ‘situation’ offers a productive way to think of the ‘transactional’ inseparability between organism and environment (see also Gallagher 2017, 54–55).

What follows from our previous considerations is that in the case of human becoming there is both continuity and discontinuity. The choice to emphasise human separateness over continuity with the rest of the animal world (and vice versa) carries no a priori epistemological value. The challenge is how we go about identifying the differences (or similarities) that make a difference within this broad comparative biological and evolutionary context. One traditional way to tackle this problem has been to differentiate between differences in degree and those in kind. As Darwin rightly insisted: ‘The difference in mind between man and the higher animals, great as it is, is certainly one of degree and not of kind’ (Darwin 1871, 105). But so far as the human entanglement with material culture is concerned, Darwin’s claim is rather unhelpful. Reaffirming this Darwinian insight seems important but, in itself, does not necessarily bring us any closer to understanding the changing meaning and impact of technical mediation in human evolution. The lack of conceptual clarity about the use and meaning of terms such as degree, kind, mind and tool use is a major contributing factor for our troubles with the task of distinguishing what are the continuities or the differences that *matter* (Malafouris 2010b, 2012a, 2013).

Even within the relatively narrow and well-studied context of tool making and tool using, there is no simple or straightforward way to tell what is really the same or different between what the chimpanzee does when he cracks a nut and what early hominins did when they detached a flake from a core (see e.g. Bril et al. 2015). Assuming a priori continuities or discontinuities between tool use in early hominins and chimpanzees is not going to help us a great deal with actually discerning the similarities and differences between skills involved in stone-flaking and nut-cracking. Instead, more fruitful would be to start from a material engagement perspective seeking to understand the material constraints, their impact on agent’s actions and movements as well as on the nature of skills involved in both tasks. Both material engagement and postphenomenology recognise that affordances and resistances of materiality arise from relational use contexts (Malafouris 2008b, 2010a, b; Malafouris and Renfrew 2010; Malafouris and Koukouti 2017; Renfrew et al. 2008; Ihde 2016).

Human intentionality seems to offer a basic criterion of differentiation. Certainly, the sense of *aboutness* characteristic of human craft and design must be very different from the relationship between an animal and the nest it builds for shelter. But we will argue in the following that if human intentionality is to offer a useful criterion of human mentality, it is not because of the ways it embodies prior planning and mental representations, but rather because of the way human intentional states are directly embodied and realised in the hybrid space of situated action (see also Suchman 2006; Malafouris 2008c; Gallagher 2017, 77).

Perhaps then it is not our notion, *Homo faber*, that is problematic but the way we approach and make sense of it through the anthropocentric separatist logic that prioritises evolutionary continuities over discontinuities and vice versa. Our suggestion is that maybe the question and the ensuing debate is framed in the wrong way. Clearly, questions of continuity or discontinuity are inherently imprecise because they presuppose that we can readily determine that there is some general fundamental difference or similarity between humans and other entities (human or non-human). This presupposes that we have some clear criteria and sufficient evidence to determine when two types of entities or processes are sufficiently different or similar to be named respectively as being ‘discontinuous’ or ‘continuous’. We suggest that any two life processes can be both continuous and discontinuous relative to some aspects. For instance, when it comes to understanding human becoming, the fact that we share almost 99% of our DNA with the common chimpanzees (*Pan troglodytes*) and bonobos (*Pan paniscus*) is as important and informative as the 1% of our difference (Tallis 2011; Marks 2003). It all depends on the exact nature of the question or issue we are trying to understand. There is no exact measure for continuity or diversity.

In any case, the interesting question is not whether human and animal tool-using abilities are continuous or discontinuous (they are clearly both), but rather, how they impact the process of evolution in different species and what makes a valuable comparative analysis. If we see continuities along the hominin line so far as the making and using of cutting tools is concerned, it is not because of the percentage of the almost 99% of DNA that we share. Rather it is because no less than 99.8% of the known 2.6 Mya (or perhaps, as noted, now 3.3 Mya) history of hominin engagement with cutting tools was spent in percussive stone tool making (Whiten 2015, 1). It is the persistent practice of percussive stone tool making that generates continuity by bringing forth a network of recurrent sensorimotor and kinaesthetic contingencies with sufficient unity, not the way genes code for traits.

Of course physiological differentiation is not to be disregarded. *Homo sapiens*, now long evolved differently than our cousins, chimpanzees and bonobos, have small molars and small jaw muscles, have lost sagittal crests and have much smaller guts than any ape relative. One anthropological theory, from Richard Wrangham (2009), relates this to differentiated eating habits, or what could be called culinary technics. Food, too, is after all material, and a cooking and preparing process a *praxis.* Today we recognise that early *hominins* may well have used complex food processing techniques and tools to make food more easily digestible than without preparation (Zink and Lieberman 2016), this before fire and cooking which then further transforms food. It has been found that human metabolism is 27% higher than apes with only 10% as much time spent chewing than apes (Gibbons 2016). ‘We are what we eat’ is a variant upon making tools and tools making us, and likely had strong evolutionary impact on shaping human bodies. And, recently, while Wrangham makes a strong case for how food preparation led to human body change since the six million years since ape/human separation, neurologist Suzana Herculano-Houzel points out that apes, too, ate a different diet with different preparation, which may well have led to the divergence of body type now noted (Herculano-Houzel 2016).

To discover what are the continuities and discontinuities that matter, we need an approach that will allow us to see and to explore, on the one hand, how different forms of materiality present and affect the bodies and the senses of different animals, and on the other hand, why and how different bodies and forms of embodiment (associated with different animals) invoke or afford certain ways of engaging and using specific forms of materiality. So, we use the term *Homo faber* to signify *difference* not in the sense of sterile exceptionalism that views human beings as a species of a different kind with a special set of pre-defined properties. Instead, we use the term *Homo faber* to signify *distinction* in the enactive anthropological sense, concerned to understand the modes of being and becoming in humans and other species.

### Beyond Mere Interaction

As we saw, careful examination of the long and multiple evolutionary history of tool-related behaviours reveals that the kind of minds we have depend on the kind of tools we make and use (the word tool here is used in its broader sense of technical mediation). In many ways, not always well understood, human intelligence is the product of fabrication as much as it is the product of Darwinian evolution by means of natural selection. The human capacity to creatively exchange energy and distribute labour between biology and culture raises important questions about the impact of material culture and the meaning of ‘technical mediation’ in the evolution of our species (cf. Latour 1990, 1992, 1993, 1999; Verbeek 2005, 2011; Wheeler and Clark 2008).

However, a persistent misconception in this context that often passes unnoticed has been to see human becoming as ontologically separated, albeit engaged in some kind of epiphenomenal dialogue or interaction with technical mediation. For instance, it is common to talk and think about evolution or to describe the co-evolution of the humans and their relevant built or natural environment as an adaptation. Darwinian evolutionary thinking sees that adaptation as unidirectional (Mesoudi 2011) whereas more recent evo-devo co-evolutionary frameworks and theories of niche construction would recognise the causal reciprocity and interaction involved (Laland et al. 2000, 2014). Still, even from such an interactive perspective, the notion of adaptation implies a process by which two or more pre-formed entities, i.e. specific organisms (human and non-human) and environments (natural and artificial), come together adapting the one to the other. This construal seems to allow the possibility of two separate processes, one of human evolution and one of technological evolution. The two processes may of course interact with and impact each other but they nonetheless remain largely separate. Moreover, according to the neo-Darwinian orthodox view of culture evolution, it is clearly conceivable that there can be a process of humanisation without technical mediation. The assumption is that tools evolve much like humans evolve, namely by means of Darwinian natural selection, and that the evolution of the former influence but is not really changing the other. In other words, there is ‘interaction’ but not ‘transaction’ or ‘interrelation’.

We argue that, ontologically speaking, the above neo-Darwinian position wrongly assumes that somehow organism and environment pre-exist their relational constitution. Both postphenomenology and Material Engagement Theory aim to overcome this problem adopting an enactive and transactional approach to the study of human evolution and the meaning of adaptation (Malafouris 2009, 2016a, b; Garofoli 2016). Postphenomenology and Material Engagement Theory react against the opposition between a ‘natural’ sphere of human speciation and a ‘cultural’ realm of ‘technological’ change. By the same token, the artefacts that often embody and actively mediate those relations are not neutral or passive but shape and transform, often in unanticipated or unintended ways, human experience (cf. Latour’s concepts of ‘script’ and ‘delegation’ (Latour 1992, 1999). An implication of that is that technological change is not always progressive, linear or in any sense controlled and pre-planned. Extension and enhancement bring about dependencies and substitutions. Human evolution in the sense of becoming is not directional but inherently creative, ongoing and thus incomplete (Malafouris 2014, 2015, 2016a, b, c).

Understanding the transformative power and potential of technical mediation, on how we live and make sense of ourselves and of the world that surrounds us, provides a point of intersection between postphenomenology and Material Engagement Theory. Moreover, recognising the primacy of creative material engagement protects us from reiterating the unhelpful nature/culture split, which often results in a separatist view of human evolution and technical or cultural evolution. Technical mediation is not something that operates in a separate ‘cultural’ realm that can be reduced, or accounted for, by means of the familiar Darwinian evolutionary principles. Instead of looking at natural selection for understanding technological change, we should be focusing on the study of the creative abilities of human consciousness, the varieties of and changing opportunities for material engagement, and the ways those processes are embedded in specific social and historical environments.

Both postphenomenology and Material Engagement Theory as research programmes are characterised by their attentiveness to the analysis of mundane things and material practices, as well as different historical manifestations of the constitutive and largely inescapable or ‘natural’ intertwining of people and things. This epistemic stance promotes a clear methodological shift toward contextual and comparative anthropological and philosophical analysis. It also promotes a novel ‘awareness’ and thus critical self-consciousness about the ontological unity of people and things. Once unity is affirmed, new attitudes about care and the politics of mediation can emerge beyond the obsolete and misleading separatist visions of a ‘technology’ free life. This does not mean that technology is something that we should take for granted in its particular historical manifestations. There is nothing inherently good or bad about a new technological development, but given the importance that they have in human life and our ways of thinking, it pays to study in more detail the specific effects they might have on us. The challenge here is not how to liberate ourselves from technology: it is how to turn technology into an instrument of liberation and critical self-consciousness.

## Beyond Interiorities and Exteriorities

Another major point of convergence between postphenomenology and Material Engagement Theory is their realisation that much of what we identify as human intelligent behaviour never happens entirely inside the head of the individual but is distributed, enacted and mediated through a variety of socio-material forms and material engagement processes. The same general premise about the extensive and transactional ontology of the relationship between the mind and the material world can also be found (in different formulations and degrees) in many recent dynamical, enactive, embodied and ecological approaches to the study of human mind in philosophy and cognitive science (Varela et al. 1991; Clark 1997, 2008; Chemero 2009; Thompson 2007; Hutto and Myin 2013; Gallagher 2017). Still, the mentioned approaches rarely emphasise enough or take into serious consideration the importance of technique and the details of material culture. Yet, there is very good potential for cross-fertilisation among those rapidly developing trends in philosophy of mind and beyond (see Malafouris 2018). Similarly, traditional phenomenological approaches with their strong emphasis on the first-personal character of consciousness sometimes obstruct a satisfactory, truly interactive and decentralised understanding of the evolving co-constitutive relationship between mind and matter. Postphenomenology and the material engagement approach share that interest in the exploration of how things matter in human thought and action and attempt to offer a rich account of the manifold ways in which humans and material objects are related to each other and in different contexts.

By the same token, we find it necessary to cast doubts upon the generally accepted cognitivist or computational principles of evolutionary psychology, also implicit in current Darwinian approaches to cultural evolution, which assume that culture can be understood as ‘information’ (in the broad sense of knowledge, beliefs, attitudes, norms, preferences and skills) acquired and stored in human brains by various mechanisms of social transmission and learning (see Mesoudi 2011; Richerson and Boyd 2005). The Darwinian processes and reasoning that has proven so useful in biology cannot be applied to the study of technics. Technics are not represented or stored inside brains. Technics are enacted by situated persons. Human–technology relations are not representational relations but embodiment relations (Ihde 1979, 1990, 2015).

This basic idea has a long heritage in philosophy. We already mentioned Bergson’s idea of ‘creative evolution’ and Dewey’s emphasis on experience, but it is with Merleau-Ponty’s phenomenological psychology, especially his publication of *the Phenomenology of Perception* (1945), that we see a radical rejection of early modern epistemology based upon the seventeenth century works of both John Locke and Rene Descartes. Theirs was a subjectivist ‘mind in a body-box’ which led to sense data, introspection and single-person inner experience. Early modern epistemology ‘invented’ the inner subject who knows only inner thought. Merleau-Ponty rejected that view—and with it also the earlier Husserlian ‘ego centered’ notion of consciousness, to lead instead to a series of descriptors revolving about being ‘outside oneself in a world.’ ‘This phenomenal field is not an ‘inner world’, the ‘phenomenon’ is not a ‘state of consciousness or a ‘mental fact’ and the experience of phenomena is not an introspection.’ (Merleau-Ponty 1962 [1945], 59) This view, which includes both ‘inner’ and ‘outer’ perspectives, is nicely illustrated in the classical example of the blind man’s cane used also by Michael Polanyi and Gregory Bateson (1972). This example of the blind person with a stick points to what is also convergent between postphenomenology and material engagement theory: Where does the blind man’s self end and the rest of the world begin? From a phenomenological perspective, it can be argued that the blind man using a stick does not sense the stick, but the presence or the absence of objects in the outside environment. Although the stick offers the actual means for this exploration, it is itself forgotten. As Merleau-Ponty describes:

‘The blind man’s stick has ceased to be an object for him, and is no longer perceived for itself; its point has become an area of sensitivity, extending the scope and active radius of touch, and providing a parallel to sight. In the exploration of things, the length of the stick does not enter expressly as a middle term: the blind man is rather aware of it through the position of objects than of the position of objects through it. The position of things is immediately given through the extent of the reach that carries him to it, which comprises, besides the arm’s reach, the stick’s range of action’ (1962, 143). Postphenomenology has taken these notions far into variants of prostheses as in Helena De Preester’s studies (De Preester 2011; De Preester and Tsakiris 2009). As with many other examples of prostheses, with time and practice the stick becomes incorporated, and thus transparent. Tactile sensation is somehow projected onto the point of contact between the tip of the stick and the outside environment. Tactility becomes a distance sense.^2^ In short, on one hand, the body schema extends to incorporate the stick, and on the other, the brain treats the stick as if it were part of the body. What about the stick? Even for those of us willing to subscribe to some of the current relational models of embodiment that recognise that differentiations between ‘inside’ and ‘outside’ often do not apply in the context of mediated activity and material engagement, the ontological status of the stick remains underspecified. Some of the most persistent questions about the emergence and evolution of human intelligence depend on precisely where one decides, implicitly or explicitly, to draw the line between the mind and the material world, and infer the direction of causality between biology and culture.

Our inherent difficulty in conceptualising the ontology of the stick largely stems from the still-dominant representational habit of imagining the mind as a brain-bound computational device. The example of the blind man with a stick aims to help us break away from those habits, and redraw the line that separates brains, bodies and things. More than a mere thought experiment, this example has been employed in the context of material engagement theory as a working hypothesis, stating that the functional anatomy of human intelligence (brain and body) is a dynamic construct remodelled in detail by behaviourally important experiences, which are mediated—and often constituted—by the use of material objects which, for that reason, should be seen as continuous, integral parts of the human mind (Malafouris 2008b, 2013). The example of the blind man’s stick offers an intuitive way of shifting our attention from the distinction of ‘mind’ and ‘matter’ or ‘in’ and ‘out’ toward developing common, relational ways of thinking about the complex interactions among brain, body and world. The transactional character of the relation between the blind man and the stick provides a diachronic point of reference for advocating an ontological continuity between mind and matter. It also helps us to re-conceptualise the profound embodiment, ecology and plasticity of the human mind.

Take for instance the tools of the Stone Age. Despite their obvious differences in terms of size, style and technique, all these tools are the products of a simple fracturing process. Archaeologists call this process ‘knapping’; the striking of a flake off a core (Roux and Bril 2005). If, as we proposed, fabrication matters in human becoming, then artefacts like these offer the starting point for our analysis. Of course, tool making represents only a small island in the sea of technical possibilities. It is the durability of stone, rather than some special status or predilection for the skill of knapping, that is largely responsible for the prominent place of stone tools in the archaeological record. Knapping stone and using stone tools was simply one among many technics or forms of material culture utilised by early humans. But leaving aside the preservation bias toward lithic artefacts in archaeology, it is a different kind of ontological bias that we found most worrying—and which we hope the example of knapping can help us expose and overcome. That is the question of the boundaries of mind.

As Lambros Malafouris points out in his *How Things Shape the Mind* (2013), the process of knapping is crucial not simply in the archaeological sense of what it can or cannot tell us about the evolution of human skill, society or technology, but also in relation to two fundamental metaphysical problems/themes that run deeply and persistently through the history of philosophy: namely, the mind–body problem and the problem of human consciousness or intentionality. Tool making makes an interesting case for metaplasticity demonstrating the complex transformations of energies and materials between the human organism and its cognitive niche.

Let us focus on one famous example, the ‘Acheulean handaxe’. Technically, it is a ‘biface’ used for butchery and woodworking between 1.5 million and 0.3 million years ago initially by *Homo erectus*. The enormous geographical distribution—ranging across Africa, the Middle East, most of Europe and large parts of Asia—and its wide temporal distribution means that it was probably the longest-lasting piece of material culture in the archaeological record (Lycett and Gowlett 2008; Ihde 2018). More interestingly however, given our purposes in this paper, is the controversy over the symmetrical (typically teardrop-shaped) form of these early bifaces. In particular, some archaeologists will see ‘conscious intention’ behind the symmetry of the handaxe morphology (for a summary discussion, see Lycett 2008; Malafouris 2010b, 2013). On the other side of the debate, many archaeologists would disagree arguing that the perceived symmetry is simply a consequence of the manufacture technique, rather than a product of human intention or conscious choice on the part of Acheulean toolmakers (Noble and Davidson 1996; Wynn 1995). Within archaeology, anthropology, and philosophy, debate over those issues remains hindered by prevalent conceptions about the mind’s location, together with a flawed ontology of material culture. The dominant ‘computational’ and ‘neo-Darwinian’ trends remain the source of confusion reiterating the idea of a mind separate from the body, a mind prominently in control of the body and a mind strangely untouched and unaltered by any of our countless interactions within the world.

One obvious implication of this metaphysical predicament is that the handaxe, a thing made of stone, cannot participate in the knapper’s cognitive realm per se. It can only be the product, or external representation, of an ‘internal’ pre-formed idea, or cognitive process, which was subsequently realised in the external physical world. Fixing ‘the marks of the cognitive’ in this traditional dualistic sense, the handaxe, like any other tool, can only be seen as a kind of epiphenomenal trace or cognitive residue left in the archaeological record by the operational sequence of the knapping gesture. The handaxe, then, offers a residual assemblage of cognitive traces from the past that an archaeologist could use as an ‘indirect’ means of *entering into* the cognitive realm proper, producing inferences about past ways of thinking. But is this ‘internal’ cognitive realm where we really need to be? And indeed, being where? Where do the mind stop and the stone tool begin? Are there sufficient grounds, beyond mere habit or convenience, to uncritically accept the dualistic representational logic of the above popular ‘internalist’ scenario?

Given the ontological commitments that both postphenomenology and material engagement theory share, this question must be answered in the negative. Accepting the internalist metaphysics of mental representations would be to deny the centrality of the lived experience of knapping as a form of embody-ing and of tools as enactive cognitive prostheses (Malafouris 2008b, 2010b). The stone in the knapper’s hand does more than passively offering the necessary ‘conditions of satisfaction’ to the knapper’s intention. The act of knapping does not simply execute the knapper’s intent already formed in the knapper’s head before the act but rather *brings forth* the knapper’s intention. The flaking intention is constituted, at least partially, by the stone itself. Information about the stone is not internally represented and processed by the brain to form the representational content of the knapper’s intentional stance. Instead, the stone, like the knapper’s body, is an integral and complementary part of the intention to knap. Every stroke prepares and carves the platform for the next. Every stroke can also reveal something new about the stone’s qualities and affordances. This by no means denies that knapping, as a form of embodied manual skill, is intrinsically associated with, follows from and leads to specific patterns of neural activation (see Stout et al. 2008). However, seeing knapping in that way avoids the usual neurocentric fallacies that take the brain as the executive controller for embodied activity; rather, it is the other way around: Now embodied activity controls the relevant activation networks of the brain. Human thought ‘stays *with* the body rather than *within* the body; it is *handmade*’ (Malafouris 2013). Intention no longer comes before action, consciousness is extensive, mind and action are one. Thus, the mental and the physical are not two opposite poles but find unity through the process of knapping. Similarly, there are no fixed agentive roles in this process; the stone projects toward the knapper as much as the knapper projects toward the stone, and together they constitute an *extended intentional state*. As Malafouris remarks:

The knapper first thinks *through* and *with* the stone before being able to think *about* the stone and hence about himself as a conscious and reflectively aware agent. In tool making, all formative thinking activity happens where the hand meets the stone. There is little deliberate planning involved (not, at least, in the sense implied in most archaeological interpretations), but there is a great deal of approximation, anticipation, guessing, and thus ambiguity about how the material will behave. Sometime the material collaborates; sometime it resists. In time, out of this evolving tension comes precision and thus skilfulness. Knapping, then, is not about externalizing pre-formed ideas or imposing form on matter. It is, instead, about learning how to make and sustain an idea and developing an explicit “sense of agency.” The knapper’s sense of agency emerges out his artificial alliance with the material hand. It is this hybrid coalition that enabled the directedness of knapping (2013, 176–77).

Ihde recognised that one should study variations on both robotic and animal capacities in order to understand the different routes embodiment can take, particularly in the contemporary proliferation of new insights from both AI and animal studies (Ihde 2015, 2018). The philosopher Peter-Paul Verbeek uses the notion of ‘cyborg’ to make a similar point about the phenomenon of human intentionality as partly constituted by technology. He introduces the term ‘cyborg intentionality’ (2008b) to express the different kinds of intentional relations between humans and technologies. Verbeek distinguishes three major forms of ‘cyborg intentionality’ to describe the different blends of human and non-human beings: First, he writes of *mediated* intentionality which is a notion originally developed in the work of Don Ihde (1990). *Mediated* intentionality is used to express the simple fact that most of the relations we have with the world around us are either mediated by or directed at technological devices and artefacts. In particular, intentionality can work *through* technological artefacts, as when we wear our glasses to read a newspaper, it can be *directed at* artefacts, as when we read the newspaper, and it can even take place *against the background* of them, as when we turn the light switch on in order to read the newspaper. The second type is *hybrid* intentionality and refers to the actual *merging*, rather than interaction, of the human with the technological. Finally, we have *composite* intentionality which Verbeek defines as an ‘addition’ or ‘interplay’ between human intentionality and the intentionality of technological artefacts themselves. In this sense, a thermometer’s directedness with respect to temperature shapes human directedness with respect to measuring temperature (2008, 387–8). Relevant here are also notions of enactive intentionality (Gallagher 2017) and of skilled intentionality, the latter expressing the tendency toward an optimal grip on a situation by being selectively responsive to multiple available affordances simultaneously (Bruineberg and Rietveld 2014; Rietveld and Brouwers 2017).

## Conclusion

Since early prehistory, we humans have been shaping our minds, constituting and reinventing ourselves through the stuff we make and the skills we develop in using them. This emphasis on technical mediation and material engagement is what unites the perspectives of postphenomenology and Material Engagement Theory (MET).

The notion of *Homo faber* has been used in this paper to signify this crucial aspect of human becoming. We have tried to re-approach the notion *Homo faber* in a way that, on the one hand, retains the power and value of this notion to signify the primacy of making or *creative material engagement* in human life and evolution and, on the other hand, reclaims the notion from any misleading connotations. Our main thesis has been that fabrication lies at the heart of the human condition. This is not an argument for human exceptionalism (other animals make and use tools). It is also not an argument for or against continuity between human and animal tool-using abilities (no animal makes and uses tools the way humans do). We have argued that we are *Homo faber not just because we make things but also because we are made by them*. People are both changing and changed by technology. The argument we have sought to develop is not one favouring technological determinism or utopianism but one that emphasises the active role of material engagement in the enactment and constitution of human life. Materiality and the forms of technical mediation that humans make and use are not passive or neutral but actively shape what we are in a given historical moment. The challenge for us is understanding in which ways and to what degree human beings are shaped and constituted by the stuff they make. Why do humans *care* so much about things? What are the implications of that for our understanding of human becoming?

Answering those questions demands cross-disciplinary collaboration that takes into account the evolutionary, historical, social, moral and political effects of technology. More important than the sheer quantity, increasing variety and dependency on material stuff in our lives is the profound complexity of our engagement with them. The devil is in the details. A superficial reading of what is at issue and lack of a genuine cross-disciplinary understanding of what is at stake could easily lead to a ‘soft’ version or understanding of material engagement. Such a ‘soft’ version has a certain appeal because it seems compatible with current neo-evolutionary and cognitivist paradigms demanding few, if any, amendments to their major postulates. But it would in fact change very little. We advocate a more ‘radical’ approach and we have tried in this paper to make explicit some of the implications that such an approach has for our understanding of human becoming. We proposed that human becoming can be accounted better by means of technical mediation and *creative material engagement* than by means of Darwinian evolution and natural selection. Failure to see this basic point has been the source of much confusion among the disciplines responsible for delineating the shape of human evolution and for updating our understanding of what it means to be human.

## Footnotes

1 Material Engagement Theory, focusing primarily on skills and material prostheses from the past such as stone knapping (Malafouris 2010b, 2012a, 2013; Iliopoulos and Garofoli 2016; Roberts 2016; Overmann and Wynn 2018; Roberts 2016; Walls and Malafouris 2016), body ornamentation (Malafouris 2008a,b; Iliopoulos 2016, 2017), image making (Malafouris 2007), pottery making (Malafouris 2008c, 2011, 2013; Malafouris & Koukouti 2017; Arnold 2018; March 2017), writing (Malafouris 2012b, 2013), numerical thinking (Malafouris 2010b, 2013; Overmann 2016a,b, 2017) and postphenomenology often focusing upon recent skill transformations and prostheses (from computer games to micro surgery to science and art) (De Preester 2009; 2011; Ihde 2016)

2 Merleau-Ponty’s analysis of technical mediation in the blind man’s stick takes a highly remote turn in contemporary prosthetic technologies. Both Ihde and Rosenberger have addressed remote sensing technologies (Ihde 2008; Rosenberger 2011, 2013) and Ihde has frequently addressed the practices of computer game playing as a pre-skilling practice which today helps those skilled at ‘Nintendo surgery,’ i.e. the eye-hand skills which are useful for laparoscopic surgical technologies have now often dominated doctors dealing with auditory, angioplasty and other micro-surgery (Ihde, Henry Lowood and Raiford Guins, *Debugging Game History (*MIT Press, 2016, pp. 127–132). Similarly, remote sensing, such as the Mars Explorer and most drone controlling, relies on simulation pre-training (Rosenberger, 2011). Many other contemporary case studies are collected in the Lexington Books series (Ihde et al. 2015; Botin et al. 2015). Cell phones, media technologies and other contemporary technologies are included.
